# Engagement with perinatal mental health services: a cross-sectional questionnaire survey

**DOI:** 10.1186/s12884-019-2320-9

**Published:** 2019-05-14

**Authors:** Alice Ayres, Renee Chen, Tracey Mackle, Emma Ballard, Sue Patterson, George Bruxner, Alka Kothari

**Affiliations:** 1 Child and Youth Mental Health Service, Child Health Queensland, South Brisbane, Queensland 4010 Australia; 2 Metro North Mental Health Service, Brisbane, Queensland Australia; 3 Redcliffe Hospital, Brisbane, Australia; 4 Faculty of Medicine, University of Queensland, Brisbane, Queensland Australia; 5 Faculty of Health and Behavioural Sciences, University of Queensland, Brisbane, Queensland Australia; 6 School of Dentistry, University of Queensland, Brisbane, Queensland Australia; 7 Queensland Institute of Medical Research, Berghofer Medical Research Institute, Brisbane, Queensland Australia

**Keywords:** Patient engagement, Perinatal care, Depression, Anxiety, Barrier, Facilitator

## Abstract

**Background:**

Perinatal depression and/or anxiety disorders are undertreated pregnancy complications. This is partly due to low rates of engagement by women. This study aimed to identify barriers and facilitators to women accessing perinatal mental health services in an outer metropolitan hospital in Queensland, Australia.

**Methods:**

Data was collected from pregnant women through a cross-sectional survey. Women rated the extent certain factors influenced their engagement. Respondents were separated into three groups: women who were not offered a referral to perinatal mental health services, women who were offered a referral but did not engage, and women who engaged.

**Results:**

A total of 218 women participated. A response rate of 71% was achieved. 38.1% of participants did not believe themselves knowledgeable about mental illness in the perinatal period, and 14.7% did not recall being asked about their mental health during their pregnancy. Of those participants who recalled being asked about their mental health, 37.1% were offered a referral. Of these, just over a third (36.2%) accepted, and out of this group, 40% attended an appointment. Regardless of referral and engagement status, the factors identified as influencing participant engagement were time restraints, lack of childcare support, and encouragement by family and health care professionals. Stigma was not identified as a barrier.

**Conclusions:**

Perinatal mental health service engagement could be improved by health services: ensuring universal screening and actively engaging women in the process: assisting with childcare; improving appointment immediacy and accessibility; and educating health care professionals about their influence on women’s engagement.

**Electronic supplementary material:**

The online version of this article (10.1186/s12884-019-2320-9) contains supplementary material, which is available to authorized users.

## Background

Mental illnesses are among the most common morbidities affecting women during pregnancy and the post-partum period (the perinatal period) [1]. Antenatal depression and anxiety are particularly common, affecting up to one in ten, and one in five women respectively [2–4]. Antenatal women experiencing depression are at increased risk of obstetric complications including preterm birth, low neonatal birth weight, gestational hypertension, and perinatal infant and mother mortality [5]. Psychiatric morbidity is a leading contributing cause of maternal death in Australia, with approximately one in six maternal deaths due to suicide [6, 7]. While effective treatment is available, women experiencing perinatal depression and/or anxiety (PD/A) are often reluctant to access mental health services and/or disclose mental health symptoms despite regular contact with health services [8, 9].

Research suggests that approximately half of women who screen positive for PD/A attend for a specialist mental health assessment [8]. Studies in high-income countries, predominantly in the United States of America (USA) and the United Kingdom, have identified factors affecting engagement with perinatal mental health services (PMHS). These factors include time constraints, perceived stigma, cultural implications, women’s inability to identify their symptoms, and the attitudes of family, friends, and healthcare professionals (HCP) [10–13]. Due to substantial contextual differences that exist between healthcare systems, the applicability of these identified factors in the Australian context requires further exploration. For example, in contrast to the USA, Australia provides free universal healthcare, and Australian national guidelines and policy requires health care facilities to complete mandatory universal screening for anxiety and depression during the perinatal period. Moreover, this policy has supported public education campaigns to increase public awareness of PD/A [14–16].

To date, there have been few studies examining factors influencing women’s engagement with PMHS in Australia (summarised in Table 1). This study aimed to increase engagement with PMHS by identifying modifiable barriers and facilitators to women accessing PMHS following a referral from their antenatal obstetric service.

**Table 1:** Summary of appraised studies that investigated barriers and facilitators to engagement with PMHS

Author, year, country	Study type, sample	Main findings
Bilzta et al. 2010 [10] Australia	Qualitative study by focus groups, *n* = 40 postpartum women	Findings suggest the lived experience of postnatal depression and associated attitudes and beliefs result in significant barriers to accessing help. Eight theme clusters were identified: expectations of motherhood; not coping and fear of failure; stigma and denial; poor mental health awareness and access; interpersonal support; baby management; help-seeking and treatment experiences and relationship with health professionals.
Button et al. 2017 [17] United Kingdom	Metasynthesis of 24 studies	Three main themes affecting women’s decision to seek help for perinatal distress: identifying a problem, the influence of healthcare professionals, and stigma.
Byatt et al. 2012 [18] United States	Qualitative study by face-to-face interview, *n* = 4 groups of perinatal health care professionals	Participants identified patient-, provider- and system-level barriers and facilitators to addressing perinatal depression. Provider-level barriers included lack of resources, skills and confidence needed to diagnose, refer and treat perinatal depression. Limited access to mental health care and resources were identified as system-level barriers.
Byatt et al. 2015 [9] United States	Systematic review of 17 studies	Higher rates of mental health care use were associated with implementation of additional interventions, including resource provision to women, perinatal care provider training, on-site assessment, and access to mental health consultation for perinatal care providers compared to screening alone.
Dennis et al. 2006 [11] Canada	Systematic review of 40 qualitative studies	A common help-seeking barrier was women’s inability to disclose their feelings, which was often reinforced by family members and health professionals’ reluctance to respond to the mothers’ emotional and practical needs. The lack of knowledge about postpartum depression or the acceptance of myths was a significant help-seeking barrier and rendered mothers unable to recognize the symptoms of depression. Significant health service barriers were identified.
Flynn et al. 2010 [12] United States	Qualitative study by semi-structured interviews, *n* = 23	Two broad themes influencing depression treatment usage emerged including practical and psychological factors. Among practical factors, women reported a strong preference for treatment provided in the obstetric clinic or in the home with a desire for a proactive referral process and flexible options for receiving treatment. Psychological factors included differing conceptualizations of depression, knowledge about severity and treatment and issues of stigma.
Goodman 2009 [13] United States	Quantitative study by cross sectional survey, *n* = 509 antenatal women	The greatest perceived potential barriers to treatment were lack of time (65%), stigma (43%), and childcare issues (33%). Most women indicated a preference to receive mental health care at the obstetrics clinic, either from their obstetrics practitioner or from a mental health practitioner located at the clinic.
Highet et al. 2014 [19] Australia	Qualitative study by interview, *n* = 28 postpartum women	Particular symptoms of anxiety and depression develop in the context of the numerous changes inherent to the transition to motherhood and contribute to a common experience of frustration and loss. Symptoms were also associated with feelings of dissatisfaction with the pregnancy and motherhood experience.
Kim et al. 2010 [8] United States	Mixed methods approach by telephone interview, *n* = 51 perinatal women	Barriers to successful treatment linkage were identified at the patient, provider, and system levels. Although 59% of at-risk women accepted mental health referrals, only 27% ultimately engaged in treatment.
Kopelman et al. 2008 [20] United States	Mixed methods approach, *n* = 1416 antenatal women	Results suggest that addressing financial and logistical barriers through changes in mental health services and policy will improve access to care for antenatal depression.
McCarthy. 2008 [21] Australia	Qualitative study by interview, *n* = 15 postpartum women	The majority of women interviewed had reached “crisis point” before they sought and received treatment. The stigma attached to an inability to cope and being a “bad mother” emerged as the main barrier to seeking help earlier. In addition, women were unable to differentiate between “normal” levels of postpartum distress and depressive symptoms that might require intervention. Talking about their distress and experiences, both with health professionals and other mothers, was regarded as of primary importance in the recovery process.
Myers et al. [22] 2013 United States	Systematic review of 40 studies	Rates of referral and treatment for women with positive screening results were substantially higher in two studies where screening, diagnosis, and treatment were provided in the same setting.
Myors et al. 2014 [23] Australia	Mixed-methods study, *n* = 244 perinatal women	Results indicated there was no significant difference in the risk factors for mental illness during the perinatal period in women who engaged and those who did not with PMHS. The time lag between initially assessment and contact by PMHS was a barrier to initial engagement. Stigma was another barrier and clinicians using women led model of service delivery with flexibility was more likely to be successful to promote engagement.
Reilly et al. 2013 [24] Australia	Case control study, *n* = 1804 drawn from the Australian Longitudinal Study on Women’s Health	The odds of receiving a referral were up to 16 times greater for women who were asked about both their past and current mental health than for women who did not receive any form of mental health assessment.

## Methods

### Setting

The study was conducted at a 270-bed public outer metropolitan hospital (OMH) serving a socio-economically disadvantaged region in Queensland, Australia [25]. On the outskirts of the capital city, in a ‘growth corridor’, the catchment population of ~ 151,000 has relatively higher rates of hospital utilisation than Queensland residents generally [26].

Pregnant women residing in the OMH catchment can access free obstetrician-led outpatient antenatal care, regardless of their pregnancy risk profile. At the initial visit (7–14 weeks), service midwives routinely screen patients for symptoms of depression and anxiety, using the Edinburgh Postnatal Depression Scale (EPDS). As part of this process, women are provided written and verbal information about PD/A. Women who score ≥ 13, and/or have a positive response to the item (ten) regarding thoughts of self-harm, are offered a referral to the local PMHS. When a referral is received, the PMHS arranges outreach assessment or appointments at the OMH. Engagement with PMHS is voluntary. Practice-based evidence (Mackle 2016, personal communication, 5th June) indicates that one-third of women who are referred to the local PMHS agree to make an initial appointment, and of these women, only one-third attend.

### Ethics approval

The study was approved by the authorised certified Human Research Ethics Committee (HREC/16/QPCH/237) and the site Research Governance office of the hospital. Before accessing the questionnaire, potential participants were provided verbal and written information by the study investigators and advised that completion of the questionnaire would imply consent for the use of the data for research purposes.

### Design

A cross-sectional survey was conducted using a questionnaire designed specifically for this study. The questionnaire (additional file 1) was iteratively developed in several stages. Initially, a pool of items was generated based on a review of the literature (summarised in Table 1) and consultation with obstetricians, midwives, mental health clinicians, and psychiatrists with expert knowledge of the population of interest. The first section of the questionnaire contained items concerning participant demographic data, and their obstetric and mental health histories, specifically addressing depression and anxiety. The second section of the questionnaire invited participants to rate their self-reported mental health knowledge on a 5-point Likert scale and enquired as to their PMHS referral status and level of engagement. The third section of the questionnaire included items identifying potential barriers and facilitators to PMHS engagement. There were 22 items (11 items each for barriers and facilitators) and women were asked to rate, on a 3-point Likert scale, the extent to which these factors had influenced or would have influenced their engagement with PMHS. There was no free text option available and the questionnaire took a maximum of 5 minutes to complete.

**Fig. 1:**
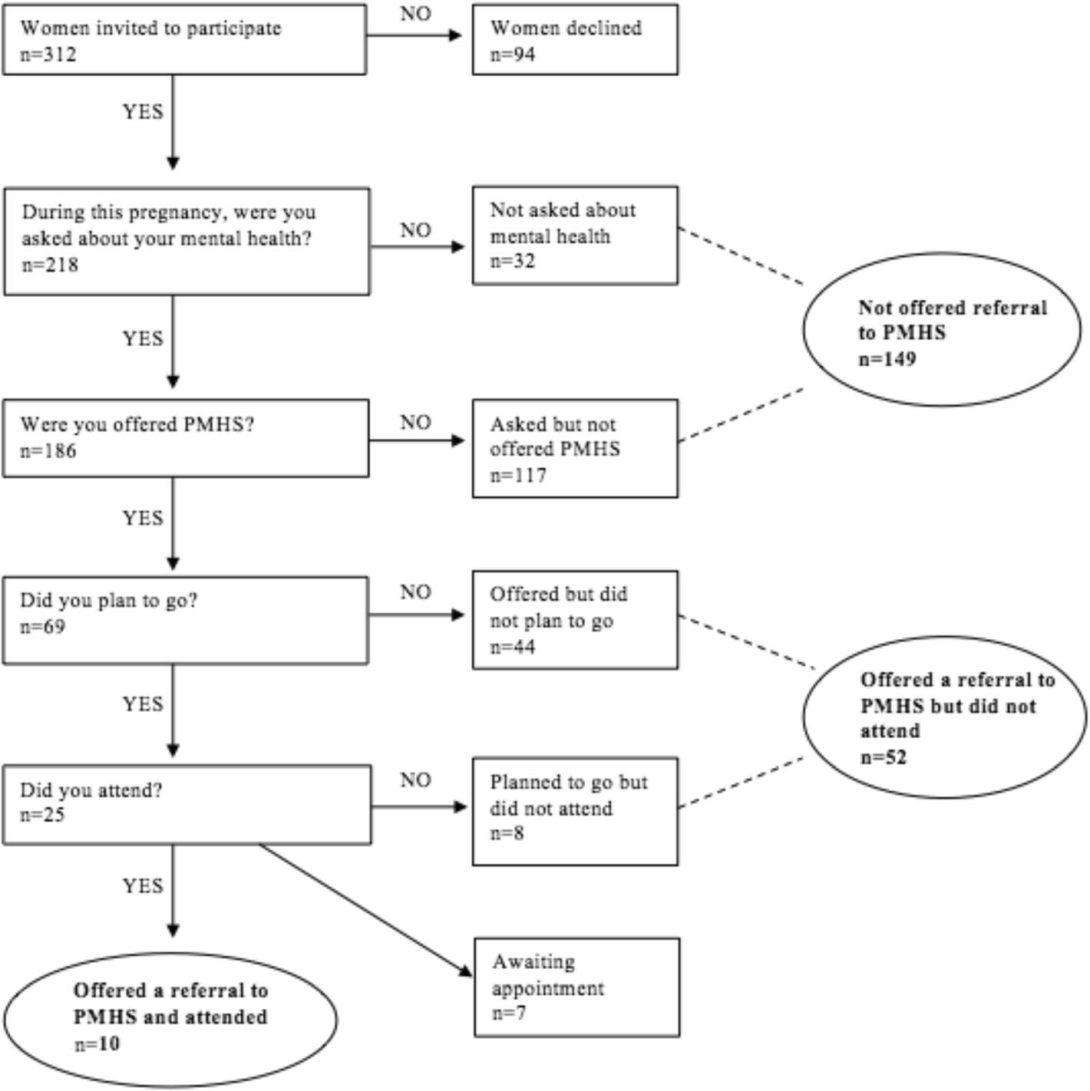
Tree algorithm implemented in study design

A tree algorithm was utilised for the questionnaire, as depicted in Fig. 1. The three primary branches of the questionnaire were tailored for (1) *women not offered referral to the PMHS (WNOR)*; (2) *women offered referral to the PMHS who did not attend (WDNA)*; and (3) *women who were offered a referral to the PMHS and attended (WA)*.

The questionnaire was piloted with ten women to assess face and content validity. Cognitive interviewing was used to explore interpretation and comprehensibility of each item. Adjustments were made to the general structure of the questionnaire, and the wording and sequence of items based on the feedback of this pilot group. The women on whom the questionnaire was piloted, appreciated the anonymous nature of the questionnaire and found the Google Forms format user-friendly.

### Participants

Women receiving antenatal care at the hospital were eligible for participation if they were aged 18 years or older, attending their first medical antenatal appointment, and sufficiently fluent in English to complete the consent processes and questionnaire.

### Recruitment and data collection

Recruitment for the study was time limited and occurred from February 2017 to July 2017. Convenience sampling of consecutive, unselected women attending their first medical appointment (generally ≥20 weeks gestation) were approached individually in the antenatal clinic waiting room by one of the researchers (Author 2). To prevent the perception of coercion, this researcher was not involved in the provision of healthcare to the individual, and women were advised their health care would not be influenced regardless of their decision to participate. Prior to the medical appointment women would typically have been reviewed by a midwife, completed the EPDS, provided education about PD/A and referred to PMHS if appropriate. After being provided patient-oriented verbal and written information about the study by the approaching researcher, women who met eligibility criteria and agreed to participate were provided with a portable electronic device with access to the questionnaire in Google Forms. The researcher was readily available if women had clarification questions.

### Statistical analysis

Statistical analysis was performed using STATA version 13 [27], which involved the calculation of descriptive statistics and comparisons of women by referral status.

For analysis, the Likert scale was collapsed into dichotomous categories for the 2 items related to knowledge of mental health: Not knowledgeable = 1, 2, 3 and Knowledgeable = 4, 5. For the items related to barriers and facilitators, responses were also collapsed into dichotomous categories of “Not at all” and “A little”/“A lot”. The “Not applicable” response was converted to missing. The frequencies of responses for these items were concentrated at the extremes of the 3-point scale. Dichotomous categorisation of the responses for these items focused on improving the interpretability of the data.

Responses to section three of the questionnaire for women who were still waiting for their PMHS appointment and planned to attend (*n* = 7) were excluded from the analysis, as it was impossible to determine if they would have engaged with PMHS.

Responses to each question were summarised by frequency and percentage. Categorical variables were examined using the Pearson Chi-squared test, or Fisher’s Exact test when more than 20% of the expected values were less than five.

## Results

Questionnaires were completed by 218 of the 307 women approached giving a response rate of 71%. Participant characteristics are described in Table 2. The majority of participants (83.9%; *n* = 183) were aged less than 35 years; with 24.3% (*n* = 53) aged 18–24 and 59.6% (*n* = 130) aged 25 to 34. Around half (50.9%; *n* = 111) identified as Caucasian and 5% (*n* = 11) identified as Aboriginal or Torres Strait Islander. Just over a quarter (28.4%) of women reported that this was their first pregnancy.

**Table 2:** Participant demographics

Question	Overall (*n* = 218)
How old are you?	
18 to 24	53 (24.3%)
25 to 34	130 (59.6%)
35 or above	35 (16.1%)
Do you identify as any of the following?	
Caucasian/white	111 (50.9%)
Australasian	39 (17.9%)
Indigenous Australian	11 (5.0%)
Other	57 (26.1%)
Which of these best represents your relationship status?	
Single/Separated or divorced	25 (11.5%)
De facto/Married/Partnered but not living together	193 (88.5%)
Which of these best represents your level of education?	
High school or equivalent	102 (46.8%)
Post high school	116 (53.2%)
Which of these best represents your employment status?	
Unemployed	96 (44.0%)
Employed	122 (56.0%)
Which of the following is the best estimate of your household income?	
$0 -$37,000	59 (27.1%)
$37,001 - $87,000	99 (45.4%)
$87,001 and over	60 (27.5%)
Is this your first pregnancy?	
No	156 (71.6%)
Yes	62 (28.4%)
Was this a planned pregnancy?	
No	92 (42.2%)
Yes	126 (57.8%)

Table 3 describes the participants’ self-report of their mental health history and knowledge of mental illness. Almost half (46.8%; *n* = 102) did not consider themselves to be knowledgeable about PD/A. Almost a third (28.4%; *n* = 62) reported not being provided information on PD/A, and 14.7% (*n* = 32) did not recall being asked by a HCP about their mental health during this pregnancy. More than one third (37.6%; *n* = 82) self-reported being diagnosed with depression or anxiety at some time. This is in concordance with a higher than average incidence of self-reported anxiety and depression in a previous study by some of the authors [28].

**Table 3:** Participant understanding of mental health illness and history of depression and/or anxiety

Question	Overall (*n* = 218)
Has a health professional ever diagnosed you with clinical depression or anxiety?	
Neither	136 (62.4%)
Anxiety	11 (5.0%)
Depression	24 (11.0%)
Both	47 (21.6%)
Have you ever been treated for depression or anxiety?	
No, I have never been diagnosed with depression and/or anxiety	123 (56.4%)
No, but I have been diagnosed with depression and/or anxiety	13 (6.0%)
Yes, I am currently being treated	23 (10.6%)
How would you rate your knowledge of mental illness generally?	
Not knowledgeable	83 (38.1%)
Knowledgeable	135 (61.9%)
How would you rate your knowledge of mental illness during pregnancy and after childbirth?	
Not knowledgeable	102 (46.8%)
Knowledgeable	116 (53.2%)
Have you been given information on perinatal depression and/or anxiety during this pregnancy?	
No/Can’t recall	62 (28.4%)
Yes	156 (71.6%)
During this pregnancy, have any health professionals asked you questions about your mental health?	
No/Can’t recall	32 (14.7%)
Yes	186 (85.3%)

Table 4 summarises the participants’ referral status and engagement with PMHS. Of the participants who recall being asked about their mental health (*n* = 186), 69 (37.1%) reported being offered a referral to PMHS. Of these participants offered a referral, roughly one third (36.2%; *n* = 25) accepted the referral, but only less than half, 40.0% (*n* = 10) actually attended. Only 1 in 7 women, who were referred actually attended the appointment.

**Table 4:** Summary of engagement with perinatal mental health services for those women who were asked about their mental health by health professionals

Question	Overall (*n* = 186)
After you were asked about your mental health, were you offered any perinatal mental health services?	
No	117 (62.9%)
Yes	69 (37.1%)
After you were offered services, did you plan to go? (*n* = 69)	
No	44 (63.8%)
Yes	25 (36.2%)
Did you attend the appointment? (*n* = 25)	
I’m still waiting to attend my appointment	7 (28.0%)
No	8 (32.0%)
Yes	10 (40.0%)

Participants with a self-reported history of mental illness were more likely than those without a history to be offered an appointment to PMHS (*p* = 0.001). Participants reporting previously being treated for anxiety or depression, were more likely, than those who had never received treatment (*p* = 0.001) to plan to attend the appointment.

Table 5 summarises the responses of participants to questions about the extent to which specified factors would influence their decision to engage with the PMHS.

**Table 5:** Factors influencing decision to engage with perinatal mental health services

Question	Not offered PMHS Overall (*n* = 149)	Offered but did not attend Overall (*n* = 52)	Offered and attended Overall (*n* = 10)	*p*-value
Lack of time				0.23
No influence	45 (33.3%)	16 (35.6%)	6 (60.0%)	
Some influence	90 (66.7%)	29 (64.4%)	4 (40.0%)	
Cannot get time off work				0.098
No influence	59 (52.2%)	25 (71.4%)	5 (71.4%)	
Some influence	54 (47.8%)	10 (28.6%)	2 (28.6%)	
No one to look after my child (ren) while I’m attending an appointment				0.064
No influence	48 (40.3%)	19 (45.2%)	6 (85.7%)	
Some influence	71 (59.7%)	23 (54.8%)	1 (14.3%)	
No transport to appointment				0.46
No influence	95 (79.2%)	35 (87.5%)	6 (75.0%)	
Some influence	25 (20.8%)	5 (12.5%)	2 (25.0%)	
How long I had to wait for the appointment				0.10
No influence	81 (62.8%)	32 (80.0%)	7 (77.8%)	
Some influence	48 (37.2%)	8 (20.0%)	2 (22.2%)	
Inconvenience attending appointment				0.33
No influence	81 (60.9%)	31 (72.1%)	6 (75.0%)	
Some influence	52 (39.1%)	12 (27.9%)	2 (25.0%)	
Costs related to going to the appointment				< 0.001*
No influence	63 (48.1%)	33 (78.6%)	7 (87.5%)	
Some influence	68 (51.9%)	9 (21.4%)	1 (12.5%)	
Previous unhelpful experience with mental health services				0.074
No influence	91 (82.0%)	30 (69.8%)	5 (55.6%)	
Some influence	20 (18.0%)	13 (30.2%)	4 (44.4%)	
Partner opposed to mental health treatment				0.048*
No influence	105 (89.0%)	42 (100.0%)	7 (87.5%)	
Some influence	13 (11.0%)	0 (0.0%)	1 (12.5%)	
Not feeling motivated				0.79
No influence	85 (68.5%)	27 (62.8%)	6 (66.7%)	
Some influence	39 (31.5%)	16 (37.2%)	3 (33.3%)	
Concern about being judged				0.16
No influence	100 (80.0%)	38 (86.4%)	6 (60.0%)	
Some influence	25 (20.0%)	6 (13.6%)	4 (40.0%)	
Worried about your mental health				0.89
No influence	74 (54.8%)	21 (51.2%)	5 (50.0%)	
Some influence	61 (45.2%)	20 (48.8%)	5 (50.0%)	
Encouragement by family				0.55
No influence	52 (39.7%)	17 (45.9%)	5 (55.6%)	
Some influence	79 (60.3%)	20 (54.1%)	4 (44.4%)	
Encouraged by midwife/GP/obstetrician				0.43
No influence	43 (33.9%)	17 (44.7%)	3 (30.0%)	
Some influence	84 (66.1%)	21 (55.3%)	7 (70.0%)	
Previous good experience with mental health services				0.16
No influence	45 (45.0%)	22 (62.9%)	4 (40.0%)	
Some influence	55 (55.0%)	13 (37.1%)	6 (60.0%)	
Required by Department of Child Services				< 0.001*
No influence	49 (51.0%)	32 (100.0%)	6 (100.0%)	
Some influence	47 (49.0%)	0 (0.0%)	0 (0.0%)	
Previously suffered from postnatal depression				0.14
No influence	54 (60.0%)	24 (66.7%)	6 (100.0%)	
Some influence	36 (40.0%)	12 (33.3%)	0 (0.0%)	
Previously suffered from other mental health issues				0.67
No influence	53 (53.5%)	19 (51.4%)	4 (40.0%)	
Some influence	46 (46.5%)	18 (48.6%)	6 (60.0%)	
An appointment time that suits me				< 0.001*
No influence	46 (36.5%)	27 (75.0%)	4 (44.4%)	
Some influence	80 (63.5%)	9 (25.0%)	5 (55.6%)	
Wanting to discuss medications				0.083
No influence	63 (55.8%)	26 (74.3%)	7 (77.8%)	
Some influence	50 (44.2%)	9 (25.7%)	2 (22.2%)	
Wanting support/counselling				0.14
No influence	53 (43.1%)	23 (60.5%)	5 (55.6%)	
Some influence	70 (56.9%)	15 (39.5%)	4 (44.4%)	
Wanting to know what help is available				0.006*
No influence	52 (41.6%)	27 (71.1%)	4 (44.4%)	
Some influence	73 (58.4%)	11 (28.9%)	5 (55.6%)	

*p-value < 0.05

Comparison of the factors influencing a woman’s decision to engage with PMHS between groups indicated that *WNOR,* compared to *WDNA and WA,* reported they were hypothetically more likely to be influenced by the cost related to going to the appointment (*p* < 0.001), and whether it was required by the Department of Child Services (*p* < 0.001). Whereas, *WDNA* were less influenced by their partner opposing mental health treatment (*p* = 0.048), an appointment time that suited them (*p* < 0.001), and wanting to know what help was available (*p* = 0.006).

For *WDNA* a lack of time, no one to look after children, and encouragement by family and HCP were identified as the primary factors that influenced their decision to not engage with PMHS. In this subpopulation, over 50.0% of participants reported these factors had “some influence” on their engagement. In *WNOR* the same factors were rated as likely to hypothetically influence their engagement with PMHS. Compared to *WDNA and WNOR*, fewer *WA* reported that a lack of time, no one to look after children and encouragement by family as factors influencing their decision. However, it is noted that this is not statistically significantly different from the other two groups.

## Discussion

### Main findings

This study illustrates the low levels of engagement with PMHS in a high-risk population with a high incidence of self-reported history of anxiety and depression and emphasises the importance of identifying barriers and facilitators to provide psychiatric assessment and appropriate management. It also expands on previous research by identifying factors influencing antenatal women’s engagement with PMHS in an Australian context. These factors, regardless of women’s PMHS referral status and engagement, were: (1) lack of time; (2) no one to look after their children; and, (3) encouragement by their family and HCP. Notably, stigma was not found to be a significant barrier to engagement.

This study also identified three factors that could influence service engagement: (1) at least one quarter of women did not recall being given any information about PD/A during their pregnancy; (2) nearly 15% of women did not recall being asked about their mental health during their pregnancy; and (3) nearly half of participants did not consider themselves knowledgeable about mental illness in the perinatal period.

### Discussion and interpretation of main findings

Consistent with findings of other studies, encouragement by family members and HCP were both identified as facilitating factors to women’s decision to engage with PMHS regardless of their referral status [13, 29, 30]. As identified by Prevatt et al. [31], some women may choose not to openly disclose their emotional distress, as such HCP should proactively promote engagement with mental health services to all women and their social supports if there are identified risk factors or concerns about mental health/emotional wellbeing in the perinatal period.

In accordance with previous published literature, lack of access to appropriate childcare and a lack of time were identified as barriers to engagement with PMHS [13, 32]. Compared to Kim et al. [8], the rate of PMHS engagement in this study was low (50% vs. 24%). Button et al. [16] demonstrated that women felt they would be more likely to engage with healthcare services if childcare services could be provided. Improving immediacy and accessibility of access to PMHS could address a perceived lack of time. Byatt et al. [9] found that providing resources to women and offering on-site mental health assessments doubled the rates of engagement with PMHS compared to screening alone. Further studies identified that a short period between PMHS referral and assessment increases engagement, as some women lose interest and/or motivation [10, 18]. Therefore, health services should develop a PMHS capable of: providing timely and accessible appointments at the location of the antenatal service; offering informational resources at time of referral; and providing access to childcare for consumers attending PMHS.

The observed absence of concern about judgement as an identified barrier is inconsistent with the literature. Stigma, both from women themselves and others, has frequently been reported [11]. It has been previously established that women may choose not to disclose their distress [32], however it is unclear why stigma was not identified as a factor in this study. The exact reasons for omission are likely multifactorial and require further exploration in future studies.

A significant proportion of the cohort did not consider themselves knowledgeable about common mental illness during and after pregnancy and did not recall being screened for PD/A. Studies have consistently shown that lack of literacy on the topic of mental illness in the perinatal period impairs a woman’s ability to identify symptoms, and therefore impacts on their engagement with PMHS [10, 11, 17]. Austin et al. [14] support active education about PD/A to women, and repeated screening through the perinatal period. Due to the nature of the questionnaire, it is impossible to determine if women were screened for PD/A and/or educated about PD/A. Regardless, the results suggest that women need to be proactively engaged in a meaningful experience during the screening process, and educated on the rationale for further assessment and treatment. Goldin Evans et al. [33] and Long et al. [34] found that screening rates increase if HCP are educated about perinatal mental health, emphasizing the need for health services to increase mental health literacy amongst HCP. This would also improve health literacy amongst women and their social supports, potentially facilitating increased emotional support.

### Strengths and limitations

The cross-sectional questionnaire facilitated the concurrent collection of information about PMHS referral rates and engagement levels within the study population. Several strategies were used to engage women that were unlikely to engage with, or had refused to engage with, PMHS. The short questionnaire length and dissemination through personal invitation at the antenatal clinic provided a high response rate of 71%. Positively, women who had declined engagement with the PMHS agreed to complete the questionnaire. The anonymous nature of the questionnaire may have increased women’s willingness to answer questions truthfully.

Notably in the study sample, only 69 women were referred to PMHS and of those only 10 women had engaged with PMHS. Although the numbers were small, no significant difference was found in factors influencing this group compared to women who did not engage and women not referred to PMHS.

Limitations of the study include the inability to generalise the results to all Australian antenatal women due to self-selection of the participants through convenience sampling. There are some differences between the study population and the general population. In comparison to pregnant women in Queensland: there were a higher proportion of pregnant women under the age of 24 in the sample (24.3% vs. 19.9%); a lower rate of employment during pregnancy (56.0% vs. 68%); a higher background incidence of anxiety and depression (37.6%) and a similar proportion of women identifying as Aboriginal or Torres Strait Islander (5.0% vs 6.8%) [35, 36]. Overall, the demographic characteristics of the sample are indicative of greater socio-economic disadvantage compared to the general population, which is consistent with what is expected for the region. However, it is noted that this limits the generalisability of results obtained to a general population of pregnant women in Australia.

Regarding the analysis of results, a limitation of the study was the collapsing of responses into dichotomous categories for some items in the questionnaire. Dichotomisation of data results in the loss of precision, however due to the distribution of results being concentrated at the extremes of the scales for some items, the loss of information from dichotomisation is compensated by the benefit of improving the interpretability of results.

Women were asked to rate pre-determined barriers and facilitators, potentially resulting in loss of key themes. The EPDS was not included in the questionnaire, and therefore it is not possible to correlate the score with the level of engagement. These omissions were consciously made to decrease the length of the questionnaire and for ease of completion.

## Conclusion

In Australia, one in six maternal deaths are due to suicide [6]. Despite a clear need for mental health support during the perinatal period, engagement of women with PD/A remains a significant challenge. The following health service practice and policy implementations could improve PMHS engagement: (1) offering women attending appointments assistance with childcare; (2) providing PD/A informational resources at time of referral; (3) improving immediacy of and accessibility to PMHS through engagement at the antenatal service; (4) providing ongoing education to all HCP involved in the care of women in the perinatal period about perinatal mental health and the positive influence HCP have on women’s engagement with PMHS; (5) active engagement of women in the screening process for PD/A, and using every encounter with HCP as an opportunity for education and provision of resources to women and their social supports; (6) ensuring universal screening and repeating this process for PD/A throughout the perinatal period.

Further research into factors affecting engagement with PMHS in the Australian context is required. Qualitative studies could examine the identified factors in more detail, and identify new themes affecting engagement. Furthermore, studies evaluating the impact of the suggested service and policy implementations on engagement rates are required.

## Additional file

Additional file 1: Engagement with perinatal mental health services: questionnaire. (PDF 2252 kb)

## Abbreviations

EPDS: Edinburgh postnatal depression score
HCP: Healthcare professionals
PD/A: Perinatal anxiety and depression
PMHS: Perinatal mental health service
WA: Women who were offered a referral to the PMHS and attended
WDNA: Women offered referral to the PMHS who did not attend
WNOR: Women not offered referral to the PMHS
