# Supplementary material for: Engagement with perinatal mental health services: a cross-sectional questionnaire survey
Source: BMC Pregnancy Childbirth. 2019 May 14;19:170. doi: 10.1186/s12884-019-2320-9 (PMC6518724; doi:10.1186/s12884-019-2320-9)
Supplement: Supplementary file 1 — Engagement with perinatal mental health services: questionnaire. (PDF 2252 kb) [file 12884_2019_2320_MOESM1_ESM.pdf]

# Mental health care during pregnancy

The purpose of this survey is to gather information on the perceptions, preferences and views of women on mental health care during pregnancy. This research will hopefully help improve the delivery of mental health care to women in the future. This survey will take up to 5 minutes. Your participation would be greatly appreciated.

If you choose to proceed with the survey, this will be taken as your consent to participate in this study. The information you provide will be de-identified, which means that it cannot be traced back to you.

* Required

1. **Have you completed this survey before?** *

   *Mark only one oval.*

   - Yes *Skip to "Thank you for completing this survey.."*
   - No

2. **How old are you?** *

   ____________________

3. **Do you identify as any of the following?** *

   *Mark only one oval.*

   - Australasian
   - Caucasian/white
   - Asian
   - Pacific Islander
   - Maori
   - African
   - Latino or hispanic
   - Indigenous Australian
   - Arab
   - Jewish
   - African-American
   - Irish
   - Scottish
   - Welsh
   - German
   - Chinese
   - Dutch
   - Multiracial
   - Other

4. **Which of these best represents your relationship status?** *
*Mark only one oval.*

- Single
- Married
- De facto
- Partnered but not living together
- Separated or divorced
- Widowed

5. **Which of these best represents your level of education?** *
*Mark only one oval.*

- Under Grade 10 or equivalent
- Completed high school
- TAFE or vocational studies
- Bachelors degree
- Post graduate studies

6. **Which of the following is your main source of income?** *
*Mark only one oval.*

- Own Job
- Partner's job
- Centrelink benefits
- Money from family or friends
- Other

7. **Which of these best represents your employment status?** *
*Mark only one oval.*

- Full time
- Part time
- Casual
- Not currently employed, looking for work
- Not currently employed, not looking for work

8. **Which of the following is the best estimate of your household income?** *
*Mark only one oval.*

- $0 - $18,200
- $ 18,201 - $37,000
- $37,001 - $87,000
- $ 87,001 - 180,000
- $180,001 and over

9. **In terms of your financial situation, do you believe you are:** *
*Mark only one oval.*

- Struggling to keep up with the costs of living
- Comfortable meeting the costs of living
- Keeping up with the costs of living with extra money

10. **Is this your first pregnancy?** *
*Mark only one oval.*

- Yes
- No

11. **Was this a planned pregnancy?** *
*Mark only one oval.*

- Yes
- No

12. **If this is not your first pregnancy, how many times have you been pregnant? Ignore this question if it is your first pregnancy.**
*Mark only one oval.*

- 1
- 2
- 3
- 4 or more

13. **How far are you into your pregnancy?** *
*Mark only one oval.*

- First trimester (1-12 weeks)
- Second trimester (13-27 weeks)
- Third trimester (28 weeks onwards)

14. **Did you have an assisted pregnancy? (eg. clomifene, IVF, artificial insemination?)** *
*Mark only one oval.*

- Yes
- No

15. **Has a health professional ever told you that your pregnancy is "high risk"?** *
*Mark only one oval.*

- Yes
- No

16. **During this pregnancy, have you experienced "morning sickness" which has affected your daily functioning?** *
*Mark only one oval.*

- Yes
- No

17. **During this pregnancy, have you experienced pain which has affected your daily functioning?** *

*Mark only one oval.*

- Yes
- No

18. **Who has been the main person looking after your pregnancy?** *

*Mark only one oval.*

- Midwife
- Obstetrician
- General Practitoner
- This is my first visit
- I have not attended most of my appointments

## Untitled Section

19. **Has a health professional ever diagnosed you with clinical depression or anxiety?** *

*Mark only one oval.*

- Neither
- Depression
- Anxiety
- Both

20. **Have you ever been treated for depression or anxiety?** *

*Mark only one oval.*

- Yes, I am currently being treated
- Yes, I have been treated but not anymore
- No, but I have been diagnosed with depression and/or anxiety
- No, I have never been diagnosed with depression and/or anxiety

21. **How would you rate your knowledge of mental illness generally?** *

*Mark only one oval.*

| | 1 | 2 | 3 | 4 | 5 | |
|---|---|---|---|---|---|---|
| No knowledge | | | | | | Very knowledgable |

22. **How would you rate your knowledge of mental illness during pregnancy and after childbirth?** *

*Mark only one oval.*

| | 1 | 2 | 3 | 4 | 5 | |
|---|---|---|---|---|---|---|
| No knowledge | | | | | | Very knowledgable |

23. **Have you been given information on postnatal depression during this pregnancy?** *
*Mark only one oval.*

- ( ) Yes, written information
- ( ) Yes, verbal information
- ( ) Yes, both
- ( ) No
- ( ) Can't remember

24. **During this pregnancy, have any health professionals asked you questions about your mental health?** *
*Mark only one oval.*

- ( ) Yes
- ( ) No *Skip to question 28.*

## Untitled Section

25. **After you were asked about your mental health, were you offered any perinatal mental health services?** *
*Mark only one oval.*

- ( ) Yes
- ( ) No *Skip to question 28.*

## Untitled Section

26. **After you were offered services, did you plan to go?** *
*Mark only one oval.*

- ( ) Yes
- ( ) No *Skip to question 30.*

## Untitled Section

27. **Did you attend the appointment?** *
*Mark only one oval.*

- ( ) Yes *Skip to question 32.*
- ( ) No *Skip to question 30.*
- ( ) I'm still waiting to attend my appointment *Skip to question 30.*

28. **Please rate how much each of these factors would influence your decision to engage with perinatal mental health services:** *

*Mark only one oval per row.*

| | Not at all | A little | A lot | Not applicable |
|---|---|---|---|---|
| Lack of time | | | | |
| Cannot get time off work | | | | |
| No one to look after my child(ren) while I'm attending an appointment | | | | |
| No transport to appointment | | | | |
| How long I had to wait for the appointment | | | | |
| Inconvenience attending appointment | | | | |
| Costs related to going to the appointment | | | | |
| Previous unhelpful experience with mental health services | | | | |
| Partner opposed to mental health treatment | | | | |
| Not feeling motivated | | | | |
| Concern about being judged | | | | |

29. **Please rate how much each of these factors would influence your decision to engage with perinatal mental health services:** *

*Mark only one oval per row.*

| | Not at all | A little | A lot | Not applicable |
|---|---|---|---|---|
| Worried about your mental health | | | | |
| Encouragement by family | | | | |
| Encouraged by midwife/GP/obstetrician | | | | |
| Previous good experience with mental health services | | | | |
| Required by Department of Child Services | | | | |
| Previously suffered from postnatal depression | | | | |
| Previously suffered from other mental health issues | | | | |
| An appointment time that suits me | | | | |
| Wanting to discuss medications | | | | |
| Wanting support/counselling | | | | |
| Wanting to know what help is available | | | | |

*Skip to question 37.*

30. **Please rate how much each of these factors influenced your decision to engage with perinatal mental health services in this pregnancy:** *

*Mark only one oval per row.*

| | Not at all | A little | A lot | Not applicable |
|---|---|---|---|---|
| Lack of time | | | | |
| Cannot get time off work | | | | |
| No one to look after my child(ren) while I'm attending an appointment | | | | |
| No transport to appointment | | | | |
| How long I had to wait for the appointment | | | | |
| Inconvenience attending appointment | | | | |
| Costs related to going to the appointment | | | | |
| Previous unhelpful experience with mental health services | | | | |
| Partner opposed to mental health treatment | | | | |
| Not feeling motivated | | | | |
| Concern about being judged | | | | |

31. **Please rate how much each of these factors influenced your decision to engage with perinatal mental health services:** *

*Mark only one oval per row.*

| | Not at all | A little | A lot | Not applicable |
|---|---|---|---|---|
| Worried about your mental health | | | | |
| Encouragement by family | | | | |
| Encouraged by midwife/GP/obstetrician | | | | |
| Previous good experience with mental health services | | | | |
| Required by Department of Child Services | | | | |
| Previously suffered from postnatal depression | | | | |
| Previously suffered from other mental health issues | | | | |
| An appointment time that suits me | | | | |
| Wanting to discuss medications | | | | |
| Wanting support/counselling | | | | |
| Wanting to know what help is available | | | | |

*Skip to question 37.*

32. **After you were seen by a mental health professional at the hospital, what follow up was arranged? You can select more than one answer.** *
*Check all that apply.*

- [ ] Referral to psychologist
- [ ] Referral to psychiatrist
- [ ] Management by GP only
- [ ] Referral to other organisations (eg. Pregnancy Counselling Link, Mission Australia, Anglicare)
- [ ] Information on how to access other services
- [ ] No follow up recommendations made
- [ ] Other: ______________________

33. **How would you rate your overall experience with perinatal mental health services?** *
*Mark only one oval.*

| | 1 | 2 | 3 | 4 | 5 | |
|---|---|---|---|---|---|---|
| Terrible | ⬭ | ⬭ | ⬭ | ⬭ | ⬭ | Excellent |

34. **Did the service meet your expectations?** *
*Mark only one oval.*

- ⬭ Yes
- ⬭ No

35. **Please rate how much each of these factors influenced your decision to engage with perinatal mental health services in this pregnancy:** *
*Mark only one oval per row.*

| | Not at all | A little | A lot | Not applicable |
|---|---|---|---|---|
| Lack of time | ⬭ | ⬭ | ⬭ | ⬭ |
| Cannot get time off work | ⬭ | ⬭ | ⬭ | ⬭ |
| No one to look after my child(ren) while I'm attending an appointment | ⬭ | ⬭ | ⬭ | ⬭ |
| No transport to appointment | ⬭ | ⬭ | ⬭ | ⬭ |
| How long I had to wait for the appointment | ⬭ | ⬭ | ⬭ | ⬭ |
| Inconvenience attending appointment | ⬭ | ⬭ | ⬭ | ⬭ |
| Costs related to going to the appointment | ⬭ | ⬭ | ⬭ | ⬭ |
| Previous unhelpful experience with mental health services | ⬭ | ⬭ | ⬭ | ⬭ |
| Partner opposed to mental health treatment | ⬭ | ⬭ | ⬭ | ⬭ |
| Not feeling motivated | ⬭ | ⬭ | ⬭ | ⬭ |
| Concern about being judged | ⬭ | ⬭ | ⬭ | ⬭ |

36. **Please rate how much each of these factors influenced your decision to engage with perinatal mental health services:** *

*Mark only one oval per row.*

| | Not at all | A little | A lot | Not applicable |
|---|---|---|---|---|
| Worried about your mental health | ⬭ | ⬭ | ⬭ | ⬭ |
| Encouragement by family | ⬭ | ⬭ | ⬭ | ⬭ |
| Encouraged by midwife/GP/obstetrician | ⬭ | ⬭ | ⬭ | ⬭ |
| Previous good experience with mental health services | ⬭ | ⬭ | ⬭ | ⬭ |
| Required by Department of Child Services | ⬭ | ⬭ | ⬭ | ⬭ |
| Previously suffered from postnatal depression | ⬭ | ⬭ | ⬭ | ⬭ |
| Previously suffered from other mental health issues | ⬭ | ⬭ | ⬭ | ⬭ |
| An appointment time that suits me | ⬭ | ⬭ | ⬭ | ⬭ |
| Wanting to discuss medications | ⬭ | ⬭ | ⬭ | ⬭ |
| Wanting support/counselling | ⬭ | ⬭ | ⬭ | ⬭ |
| Wanting to know what help is available | ⬭ | ⬭ | ⬭ | ⬭ |

*Skip to "Thank you for completing this survey.."*

## Offer of help

37. **Would you like any information or assistance with your mental health?** *

*Mark only one oval.*

- ⬭ Yes *Skip to "Please see one of our staff for further information.."*
- ⬭ No *Skip to "Thank you for completing this survey.."*

## Please see one of our staff for further information.

## Thank you for completing this survey.
